# Eliminate the hardware: Mobile terminals-oriented food recognition and weight estimation system

**DOI:** 10.3389/fnut.2022.965801

**Published:** 2022-11-16

**Authors:** Qinqiu Zhang, Chengyuan He, Wen Qin, Decai Liu, Jun Yin, Zhiwen Long, Huimin He, Ho Ching Sun, Huilin Xu

**Affiliations:** ^1^Chengdu Shangyi Information Technology Co., Ltd., Chengdu, China; ^2^Sichuan Key Laboratory of Fruit and Vegetable Postharvest Physiology, College of Food Science, Sichuan Agricultural University, Ya’an, China

**Keywords:** food recognition, weight estimation, machine learning, nutrition monitoring, elimination hardware

## Abstract

Food recognition and weight estimation based on image methods have always been hotspots in the field of computer vision and medical nutrition, and have good application prospects in digital nutrition therapy and health detection. With the development of deep learning technology, image-based recognition technology has also rapidly extended to various fields, such as agricultural pests, disease identification, tumor marker recognition, wound severity judgment, road wear recognition, and food safety detection. This article proposes a non-wearable food recognition and weight estimation system (nWFWS) to identify the food type and food weight in the target recognition area *via* smartphones, so to assist clinical patients and physicians in monitoring diet-related health conditions. In addition, the system is mainly designed for mobile terminals; it can be installed on a mobile phone with an Android system or an iOS system. This can lower the cost and burden of additional wearable health monitoring equipment while also greatly simplifying the automatic estimation of food intake *via* mobile phone photography and image collection. Based on the system’s ability to accurately identify 1,455 food pictures with an accuracy rate of 89.60%, we used a deep convolutional neural network and visual-inertial system to collect image pixels, and 612 high-resolution food images with different traits after systematic training, to obtain a preliminary relationship model between the area of food pixels and the measured weight was obtained, and the weight of untested food images was successfully determined. There was a high correlation between the predicted and actual values. In a word, this system is feasible and relatively accurate for one automated dietary monitoring and nutritional assessment.

## Introduction

Poor dietary habits are closely related to many diseases, including hypertension, type 2 diabetes, coronary artery disease, dyslipidemia, various cancers, fatty liver, and obesity (1). Therefore, improving the dietary structure is the focus of solving public health problems. Most people are aware of the importance of diet for health and actively seek relevant nutritional information and corresponding health guidelines (2–4). However, simple health knowledge publicity makes it difficult to form systematic health consciousness and cognitive change; this information is not effective in preventing diet-related illnesses or helping patients eat healthily, let alone self-monitoring of diets and nutritional management; this can make it difficult to implement long-term health behaviors (5, 6). In addition, because of lacking professional nutrition knowledge, unhealthy eating habits, and poor self-control, people often ignore daily food intake and unconscious overeating behaviors (7). The comprehensive system of professional health education is the premise of improving dietary cognition, on this basis, diet measurement tools and simple and accurate nutritional assessment methods are needed to achieve personalized detection of diet and one-on-one time correction, so that cognitive changes are more implemented into the efficient implementation of behaviors (8, 9).

Therefore, there is an urgent need to provide long-term and effective solutions to help the general public and patient populations improve diet quality, food intake monitoring, and health management. At present, many researchers have tried to combine computer vision technology and deep learning neural networks with diet monitoring and health management. For example, both single food classification and fruit classification and caloric estimation are performed using digital imaging and multilayer perceptrons (10, 11). Deep convolutional neural networks can be used to estimate BMI and obesity classification (12). Build a CNN training model to classify thousands of food images and define food attributes (7). This shows that classification using computer vision technology and deep convolutional neural networks has been relatively mature.

However, the use of machine deep learning for food volume estimation is still premature; the 3D reconstruction of the food can also be performed with two views of the food to restore the shape of the food and its estimated volume; however, a marker is needed as a reference to estimate the volume (13). The current bioengineering study also proposes a new smartphone-based imaging method that can estimate the amount of food placed at the dinner table without additional fiducial markers (14). There is a food part estimation method that introduces the concept of “energy distribution” for each food image, and training a generative adversarial network (GAN) can estimate food energy (kcal) from food images (15). In addition, a food volume measurement technique based on simultaneous localization and mapping (SLAM), a modified version of the convex hull algorithm, and a 3D mesh object reconstruction technique can utilize mesh objects to calculate the volume of the target object, in contrast to previous volume measurement techniques. It can realize the continuous measurement of food volume without prior information such as pre-defined food shape models, and experiments have proved that the proposed algorithm has a high degree of feasibility and accuracy in measuring food volume (16).

In addition, wearable devices have also begun to be applied to dietary monitoring, such as the use of gesture recognition on wrist-worn smartwatches to recognize the wearer’s eating activity and monitor diet (17). Eating activity can also be automatically monitored using wearable sensors to estimate food intake (18). A microphone and a camera are combined to form a sound and video collector that is worn on the ear, and the video sequences of chewing sounds and eating activities can be obtained to realize the classification of the wearer’s food intake and the estimation of food intake (19). It is due to the complexity and diversity of food types, the accuracy of computer vision technology, and the convolutional neural network in food recognition and calorie estimation; there is no large-scale applicable dietary monitoring software and food recognition system yet (20). In addition, wearable devices are unreliable for eating behavior monitoring to estimate food intake to a certain extent, not only easy to confuse non-eating behaviors (such as smoking and nail-biting) and environmental requirements (must be in a controlled environment) but also adds additional financial burdens and increases the burden of daily behavior (18).

Therefore, we proposed a mobile-oriented non-wearable recognition and weight estimation system. To accurately identify the food in the system, we used the Aliyun Cloud Food Recognition API to accurately identify the food in 1,455 images; then we used the deep convolutional neural network and the visual-inertial system to accurately locate the food area, collect the pixels of food images, and systematically trained 612 high-resolution food images of different characters to obtain a relationship model between the area of food pixels and the measured weight; and finally, determined the weight of different food images through the relationship model. This can help the general public accurately recognize daily food types, achieve weight estimation through smartphones, and assist clinical patients and doctors in monitoring diet-related health conditions. A significant step toward automated dietary monitoring and nutritional assessment, as well as a step closer to digital nutrition therapy.

## Materials and methods

### Overall system design

**FIGURE 1:**
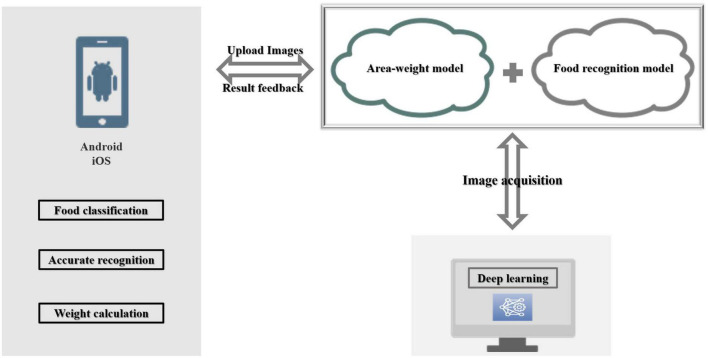
Wearable dietary monitoring system for mobile terminal.

The identification system for non-wearable food recognition and weight estimation system for mobile terminals included a mobile terminal program, an Internet computing platform, a food identification model (Aliyun food identification API), and an area-weight calculation model, as shown in Figure 1. The food database in Aliyun food identification API can be used to achieve the effect of food classification. First, food images would be acquired and imported into the mobile terminal program. Second, food images were uploaded to the Internet computing platform loaded with the food recognition model and the area-weight calculation model. Finally, performed food category recognition and weight prediction were performed.

### Food types and image data

A total of 1,455 images of different foods were taken and collected as the test sample size to verify the accuracy of the Aliyun Cloud Food Recognition API (These food images were collected by the first version of the APP after 50 volunteers were recruited and trained). Table 1 shows the sample size information for 1,455 images. According to the worldwide eating habits survey (21), 612 images of 51 common dishes were collected through mobile phone shooting (LG8 smartphone, 1.5 x, vertical view; shot in the LED studio, the size of the studio was 60 cm, 50% brightness, turn on the inside and outside lights), as shown in Table 2. Images were captured with a fixed 35 mm equivalent focal length of 46 mm, shooting height (40.6 cm), shooting angle (parallel to the horizontal plane), and a uniform background (white) to ensure consistent image quality. In addition, 612 images of 51 foods were collected for area-weight model validation using the same method as the training sample set.

**TABLE 1:** Food recognition image sample classification.

Classification of food	Sample size (n)
Overall	1,455
Meat	270
Vegetable	188
Meat and vegetable	145
Staple food	248
Fruit	134
Drink	110
Snack	113
Soup	115
Nut	66
Set meal	66

**TABLE 2:** Fifty-one common foods to model.

Block/Thick bar		Slice/Silk		Grain/Granule	
Name	Size	Dish name	Size	Name	Size
Roasted meat	12	Vinegar cabbage	12	Cucumber salad	12
Stir-fried pork with bamboo shoots	12	Shredded pork with Beijing sauce	12	Chicken diced	12
Chicken thigh block	12	Bean sprouts salad	12	Stir-fried lotus root diced	12
Sweet and sour pork ribs	12	Fried lettuce silk	12	Stir-fried pork with lettuce diced	12
Braised beans with potatoes	12	Green pepper shredded pork	12	Shrimp corn	12
Potato grilled pork ribs	12	Fried pork with garlic sprouts	12	Cowpea fleshy foam	12
Eggplant chunks	12	Stir-fried pork with mushrooms	12	Fried diced chicken	12
Pork meatballs	12	Shredded onion	12	Spicy chicken	12
Mushroom and chicken	12	Sausage slices	12	Bean curd butyl	12
Halogen pig’s feet	12	Sliced ham	12	Beef granules	12
Radish flank	12	Shredded chicken	12	Ham fried rice	12
Fried yam block	12	Vegetable salad	12	Carrot dices	12
Fried chicken wings	12	Cold noodles	12	Mushroom butyl	12
Tomato scrambled eggs	12	Needle mushroom with sauce	12	Green pepper corn	12
Konjac roast duck	12	Dried fish fillet	12	Groundnut kernels	12
Steak	12	Sliced carrot	12	Pea	12
French fries	12	Potato silk	12	Bamboo butyl	12

#### Preprocessing of recognition images

To improve the recognition rate of food, the food area was cropped from the image in a 1:1 ratio of length and width, and the image was scaled to a uniform size of 224 × 224 pixels. Because some food category images in this article were not enough to improve the generalization ability and robustness of the model, the original images were augmented by three methods to improve the generalization ability and robustness of the model: increased the image contrast and brightness, added noise, and rotated 90°, and the training set was expanded three times.

### Food recognition model

**FIGURE 2:**
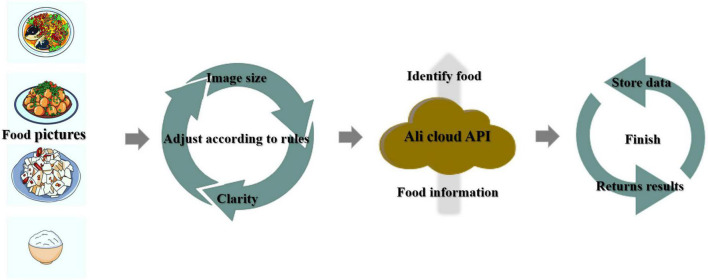
Aliyun food recognition model.

Figure 2 depicts the Aliyun Cloud Food Recognition API in use. After collecting the relevant food images, the Aliyun Cloud food recognition model was used to classify and identify the food. After the recognition was complete, the data was uploaded and saved for the next step.

### Establishment of food area-weight calculation model

#### Model building

**FIGURE 3:**
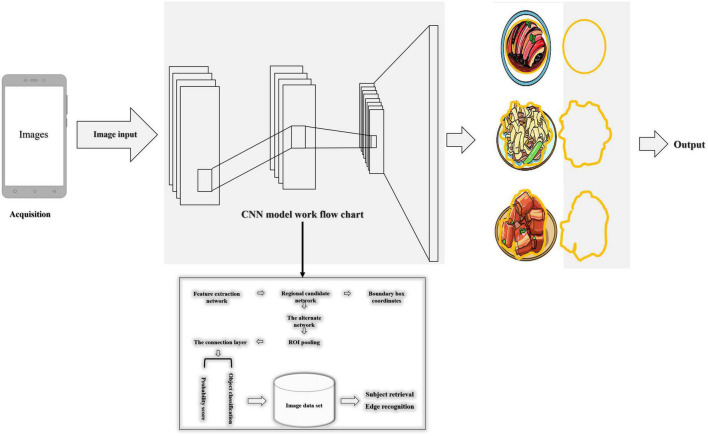
Food area-weight calculation model based on the deep convolutional neural network.

Following the use of the Aliyun food recognition model to classify and identify food, a food area-weight calculation model based on a deep convolutional neural network was established, as shown in Figure 3.

**FIGURE 4:**
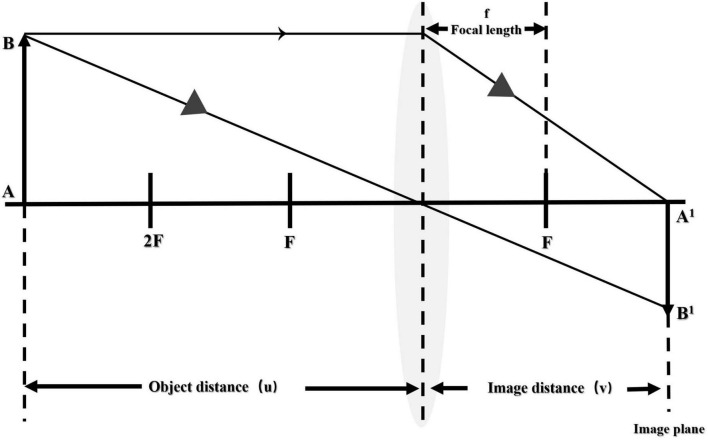
Area measured by convex lens imaging principle.

We built the following method to measure the weight of food without using other hardware than the phone. The whole method included a negative feedback adjustment algorithm before taking a picture (the acquisition of a top-view image with a certain height and focal length before taking a picture), computer vision technology, and a convolutional neural network after taking the picture to perform subject detection and edge detection to accurately locate the location and area of the food. The map’s pixel points were calculated, and the area was estimated using the convex lens imaging principle (22), a food area-weight calculation model was established, and the food weight was predicted. The main processing algorithm of the post-shooting image was to obtain food photos based on the pre-shooting algorithm environment and framed the location of each targeted subject in the image through multi-target subject detection. Target recognition was performed separately, and the food categories in the box were judged one by one. Using the principle of convex lens imaging, as shown in Figure 4, circular detection and edge detection were carried out in the rectangular frame. The circular detection determined the projected area of the tableware, and the edge detection calculated the area of the dishes by circling the range of food in the photo through a series of coordinate points.

Among them, the object distance u, the distance v, and the focal length f satisfy the following relationship: 1/v + 1/u = 1/f (23). Therefore, if you know the length of the phase, the object distance, and the focal length of the phase plane, you can measure the length of the object in the real scene. However, different cell phone cameras have different sizes of phase planes and different lengths of individual pixels. However, the equivalent focal length (rather than the true focal length) can ignore the size of the different phone phase planes or the size of the pixel unit. Because the real focal length and equivalent focal length of the mobile phone camera satisfy the following relationship:

**Table:** 

Image size	Diagonal-based EFL
4:3 (sensor width w)	*f*_35_ = 34.6 f/w mm
4:3 (sensor diagonal d)	*f*_35_ = 43.3 f/d mm
3:2 (sensor width w)	*f*_35_ = 36.0 f/w mm
3:2 (sensor diagonal d)	*f*_35_ = 43.3 f/d mm

Therefore, assuming that any image is x pixels wide and y pixels long, the resolution of the camera is a: b, the equivalent focal length is f (mm), and the object distance is u (cm), then the object in the real world; the length A (cm), width B (cm), satisfy the following relationship. If b: a is equal to 4:3, *A* = 26.0 * (x/a) * (u/f − 0.1), *B* = 34.6 * (y/b) * (u/f − 0.1).

Using a rectangular frame to lock the position of the subject of the picture through multi-target subject detection to obtain food photos based on the computer vision technology. Cut out the picture in the rectangular frame, carried out target recognition, and judged the food category in the rectangular frame. Then, in the rectangular frame, edge detection was used to circle the range of food in the photo through a series of coordinate points, and the number of all pixels in this range was calculated. Based on the object distance, equivalent focal length, number of pixels, and image resolution, the real-world food area was calculated. Assumed that the pixels circled in the image = n, then S = n*[(26.0/a) * (u/f − 0.1)]^2^, or S = n*[(34.6/b) * (u/f − 0.1)]^2^. Finally, based on the type of food and the area of the food in the real world, a machine learning algorithm was built to infer the weight of the food.

#### Preliminary validation of the model

A total of 612 images of the same dishes as in the model building set were collected using mobile phone photography. The area-weight model calculation formula (classified by traits) was used to identify the area of the food image and to calculate the weight. The effect was verified by the area-weight model estimated by the area-weight model vs. the measured weight.

### Design and implementation of mobile terminal program

#### Client development

For Android application development, an Android Integrated Development Environment (Android Studio IDE) was created on the Windows 10 platform. To exchange data with the server, use the Okhttp network transmission framework, download the third-party jar development package, place it in the lib directory, parse it into a so file after synchronization, and then request the third-party API. Furthermore, iOS is Apple’s mobile operating system that runs on iPhone, iPad, and iPod Touch hardware. If the mobile terminal program is iOS App Development, it must be developed in Apple’s integrated development environment (IDE), also known as Xcode.

#### Design and implementation of a mobile terminal program

The mobile UI interface used the bottom navigation bar style. A publicity module, a user login registration module, a photo module (image upload module), a food labeling module, a food identification module, a result generation module, a food record module, and a nutritionist/doctor monitoring module were all included in the interface.

#### Deployment of server-side recognition models

The programs of the food recognition model and the food area-weight calculation model were packaged and deployed on the server, and the mean file, weight file, and label file during the model training process were added, and the program that requests the model and outputs the recognition results was compiled into a DLL dynamic link library. When the server side receives the food image transmitted by the mobile terminal, it ran the DLL dynamic link library file. The food recognition model was first asked to recognize the uploaded image, then the food area-weight calculation model was requested to calculate the food weight, and finally, the food nutrition ingredient database was queried. After analyzing its nutritional components, the server fed back the overall analysis results to the client and saved the uploaded images and identification analysis results in the database to facilitate the traceability of food images in future.

### Statistical analysis

SPSS 21.0 (IBM, Chengdu, China) was used for data analysis and statistics. Paired *t*-test was used to analyze the statistical differences between the mean values of the data in each group, and *p* < 0.05 was considered significant. When establishing the model, Person correlation analysis was performed on the normally distributed data to obtain the correlation coefficient, and then used linear regression to establish a linear correlation model.

## Results

### Food identification model results and analysis

**FIGURE 5:**
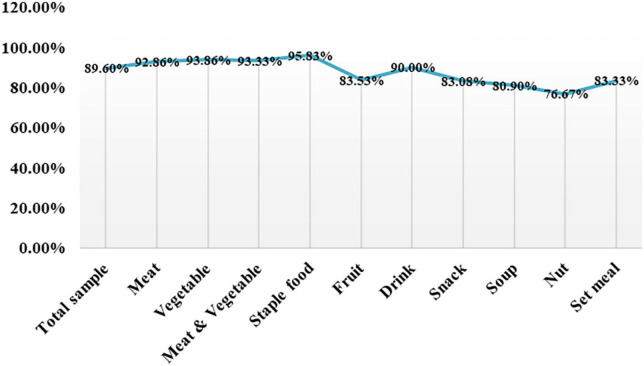
Recognition accuracy rates of the Aliyun Cloud Food Recognition API for different food classifications.

The recognition accuracy of Aliyun Cloud food identification API for 10 different kinds of food was statistically analyzed. Following Table 3 and Figure 5, the Aliyun Cloud food recognition model had a relatively high recognition accuracy rate for the 10 types of food in the table below, with the highest recognition accuracy rate for staple foods at 95.83%, and the lowest recognition accuracy rate for nut foods at 76.67%, which may be due to nuts. Its characteristics and sample size were small. The average recognition accuracy of 10 types of food is 89.60%, and the overall recognition accuracy was high.

**TABLE 3:** Recognition accuracy rates of the Aliyun Cloud Food Recognition module.

Classification of food	Sample size (n)	Recognition accuracy
Total sample	1,455	89.60%
Meat	270	92.86%
Vegetable	188	93.86%
Meat and vegetable	145	93.33%
Staple food	248	95.83%
Fruit	134	83.53%
Drink	110	90.00%
Snack	113	83.08%
Soup	115	80.90%
Nut	66	76.67%
Set meal	66	83.33%

### Food area–Weight calculation results

#### Machine-identified area–Actual area comparison

The machine food recognition area was compared with the actual area, and the paired *t*-tests found that there was a significant difference between the two (*p* < 0.05), and the machine recognition area was significantly lower than the actual area. The Pearson correlation analysis was performed on the machine identification area and the actual area. The correlation coefficient between the machine identification and the actual area was 0.979, and it showed a significant level of 0.01, which indicated that there was a significant positive correlation between the machine identification and the actual area relation. In addition, there was a strong linear correlation model between the machine-recognized area and the actual area, *y* = 0.7378x + 12.765, and *R*^2^ = 0.9591.

#### Establishment of the area-weight calculation model

**FIGURE 6:**
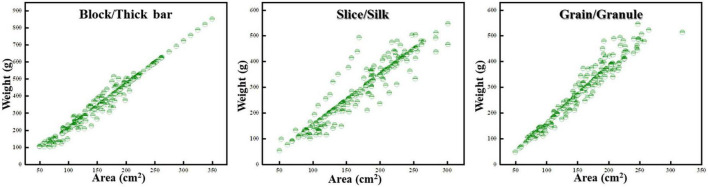
Scatter plot of food image area-actual food weight relationship.

Through the establishment of a relationship model between the area of the food image recognized by the machine and the actual weight of the food, it was found that there was a significant difference between the recognized area of the food with different traits and the measured weight (*p* < 0.05). These are shown in Table 4 and Figure 6, the correlation coefficient value between the measured weight and the block thickness-machine recognition area was 0.986 and it showed a significant level of 0.01, which indicated that there was a significant positive correlation between the measured weight and the block thickness-machine recognition area. The correlation coefficient value between the measured weight and the sheet/filament-machine identification area was 0.937, and showed a significant level of 0.01, thus indicating that there was a significant positive correlation between the measured weight and the sheet/filament-machine identification area. The correlation coefficient value between the measured weight and the grain-machine identification area was 0.972, and it shows a significant level of 0.01, which indicated that there was a significant positive correlation between the measured weight and grain-machine identification area. In addition, different traits presented different linear correlation models, as shown in the table below.

**TABLE 4:** Food image area-actual food weight relationship model.

Character	Sample size	Pearson correlation	*R* ^2^	Linear correlation formula
Block/Thick bar	204	0.986**	0.971**	*y* = 2.5757x − 49.03
Slice/Silk	204	0.937**	0.878**	*y* = 1.9684x − 46.3
Grain/Granule	204	0.972**	0.944**	*y* = 2.2069x − 62.13

*p < 0.05, **p < 0.01.

#### Validation effect of the area-weight calculation model

The food weight calculated by the machine through the area-weight model was compared with the measured weight, and the paired *t*-test found a significant difference (*p* < 0.05). At the same time, the estimated weight of the machine and the measured weight were found by Person correlation analysis. The correlation coefficient between the estimated weight of the machine and the measured weight was 0.936, which was significantly correlated at the 0.01 level. This shows that there is a significant positive correlation between the estimated weight of the machine and the actual weight. In addition, a single sample *t*-test was performed on the difference between the two groups of data, and it was found that the mean ± standard deviation was −0.017 ± 2.28, and the sig two-tailed was 0.999. The higher the consistency, the higher the accuracy of the area-weight calculation model.

### Food intelligent identification and measurement system for mobile terminals

**FIGURE 7:**
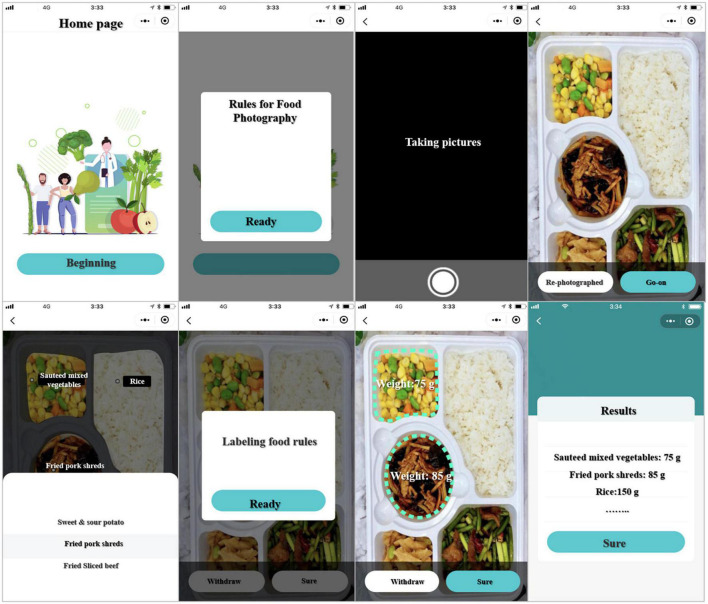
Mobile terminal application page display diagram.

The mobile terminal program was installed on a mobile phone with an Android system or an iOS system. As shown in Figure 7, there were currently eight pages.

## Discussion

Excessive calorie intake from food is already a major factor in various obesity-related diseases, increasing the risk of heart disease, type 2 diabetes, and cancer in sub-healthy people (24–26). Therefore, it is necessary to have an accurate dietary assessment system and nutritional monitoring system to achieve accurate identification and classification of daily diets and nutritional monitoring (27–29); at the same time, it can help ordinary health management users, patients, and doctors to automatically measure food intake and collect various dietary information (30). Accurate nutritional index detection is an effective way to realize disease prevention and nutritional treatment (31).

However, the current situation is not optimistic. Most clinical nutritionists and health managers still use traditional paper records and manual entry methods to measure eating behaviors, such as the 3*24 h meal review recording method, weighing method, meal frequency investigative way, etc. These traditional methods have low accuracy, complicated operations, and are prone to retrospective bias and missing data, making it difficult to achieve accurate and efficient dietary analysis (32). Therefore, the use of computer learning systems to solve this problem seems to have become a major development trend. Machine learning has shown good results in food recognition and detection and has achieved good results in food recognition, cooking method recognition, food ingredient detection, fake food image recognition, fast-food recognition, etc. (33). There are still application limitations, and there are no corresponding system tools and procedures for large-scale use (27, 34). For example, it has been reported that the use of mobile food recognition systems to identify multiple foods in the same meal can only achieve an accuracy rate of 94.11% for more than 30 types of food types, which cannot be used to estimate intake (35). In addition, the accuracy rate of most deep learning-based food recognition systems is still at the level of 50.8–88%, and the types of food involved have obvious regional characteristics and are not representative (35).

Based on the traditional machine recognition system, we optimized the computer recognition technology to achieve the accurate recognition of 1,455 food images of 10 categories of food, with an accuracy rate of 89.6%. In addition, to further realize the accurate estimation of dietary intake, the subject detection and edge detection techniques were used to accurately locate the food location and area, and after collecting the pixel points of the food area, the area was estimated using the principle of convex lens imaging. Six hundred twelve images were used for machine learning to build area-weight calculation models for different food traits. Using 612 food images for verification, it is found that the machine can estimate the weight of 51 kinds of food through the area-weight calculation model. Therefore, the method of estimating food weight by area-weight calculation model is high accuracy. This study is the first system to recognize food types and estimate food weight through food areas in dietary monitoring and nutritional assessment. Compared with other traditional dietary surveys and nutrition assessment tools, it is simple and efficient. Furthermore, it has higher accuracy and feasibility when compared to existing food recognition models, 3D reconstruction of food volume and wearable devices that monitor dietary behavior.

This indicates that food weight estimation by food image area is feasible and one of the effective ways to realize automated dietary monitoring and nutritional assessment. With the assistance of building a corresponding food nutrition database, it is expected to realize the identification of food and nutrient intake analysis by taking pictures, and finally achieve the purpose of nutrition assessment and monitoring intervention. In addition, it is worth mentioning that wearable health monitoring devices are more popular in today’s era (36). It seems more convenient and faster to build a nutrition assessment monitoring system that can be loaded on mobile phones, which can reduce the wearing process, economic burden, and living burden of wearable devices (37), with the expectation of a simpler and more efficient healthy lifestyle.

In addition, we also conduct a brief analysis of the feasibility, shortcomings, and future improvement directions of the nWFWS. It is undeniable that there are still some limitations that need to be improved. The sample size used for Aliyun Cloud food recognition model verification is not large enough, and the types of food involved are limited, containing only some common foods. Although the accuracy of food identification has reached 89.6%, a larger sample size is needed to further verify and improve the accuracy, and it is expected to achieve all-around and multi-type accurate identification of food (7, 38). The area-weight calculation model needs to be further optimized, realizing AR automatic ranging when taking pictures, reducing the requirements for taking pictures, and image processing; further expanding the sample size for training, learning, and verification, and improving more accurate weight estimation. In future, it is hoped to realize automatic photography and 3D reconstruction of food through AR, and non-contact reconstruction of food through 3D technology to measure the weight and volume of food, more accurately assess dietary intake, and more effective nutrition monitoring. In addition, although paired t-tests and personnel correlation tests are effective, they are only used to verify the effect of the initial model building in this article. It is suggested to use a loss function such as multi-class cross-entropy loss (39, 40).

## Conclusion

The food recognition and weight estimation for mobile terminals mainly include mobile terminal service programs, Internet computing platforms, Aliyun Cloud food identification models, and area-weight calculation models. When in use, you can take pictures of food according to certain shooting requirements, and use the Aliyun Cloud food recognition model to accurately classify and identify the food to achieve accurate recognition; then use computer vision technology to accurately locate the food area and range, and use the area-weight calculation model to estimate the weight of food. Measure the food in the area. The recognition accuracy rate of the system for 1,455 images of 10 categories of food can reach 89.60%, and the weight of 51 kinds of food is estimated by the area-weight model. The average difference between the estimated value and the actual value is only −0.017 ± 2.28, which has a high consistency. Therefore, this non-wearable food identification and portion estimation system can provide a promising meal record assessment and nutrition management tool for the broad self-health management population, patients, and doctors, and the system settings will be further optimized, adding clinicians/nutrition. The mobile terminal loads the food nutrient composition database realizes the “one-to-one” evaluation and monitoring record of eating behavior, and feeds back the corresponding nutritional analysis results and opinions; further realizes all-around, multi-dimensional, and efficient remote dietary monitoring and nutrition management functions.

## Data availability statement

The original contributions presented in this study are included in the article/Supplementary material, further inquiries can be directed to the corresponding authors.

## Author contributions

QZ, CH, and WQ designed the study and revised the final version to be published. QZ, DL, JY, ZL, HS, HH, and HX performed the experiments. QZ drafted, wrote, and revised the manuscript. All authors contributed to the article and approved the submitted version.
